# Assessment of Asthma Control Among Asthmatic Patients at Primary Healthcare Centers in Makkah, Saudi Arabia

**DOI:** 10.7759/cureus.11103

**Published:** 2020-10-23

**Authors:** Dalia Alansari, Tariq Abdulmalek Mirza

**Affiliations:** 1 Family and Community Medicine, King Abdulaziz Medical City, Ministry of National Guard Health Affairs-Western Region, Jeddah, SAU; 2 Family Medicine, Iskan Primary Health Care Center, Ministry of Health, Makkah, SAU

**Keywords:** asthma control, poor asthma outcomes, primary healthcare centers, saudi arabia, children, adolescents, adults, associated factors of asthma

## Abstract

Introduction

Asthma is the most common respiratory disease worldwide. In Saudi Arabia, asthma is considered as a major public health concern and has a negative impact in the life of patients, their families, and the community, including lost days of work, absence from school, and poor quality of life, which can eventually lead to frequent emergency department visits, hospitalizations, and sometimes, to death. Thus, the objectives of this study were to evaluate asthma control status among asthmatic patients in primary health care centers (PHCCs) in Makkah, Saudi Arabia and to identify factors associated with poor asthma control.

Methods

This was a cross-sectional study conducted from March to May 2016 in selected PHCCs in Makkah, Saudi Arabia. Data was collected from five PHCCs which were selected using a stratified random sample technique from a total of 47 PHCCs to represent the different geographic regions of the city. The 167 asthmatic patients, aged four years and above, presented during the study period were included. Each respondent completed two tools: the first is a self-administrated questionnaire and the second is the asthma control test. Statistical analyses were performed with SPSS version 21 software (IBM Corp, Armonk, USA). Qualitative variables were analyzed with the Chi-square test or Fisher's exact test as appropriate.

Results

Around one-third (34.1%) of all asthmatic patients were categorized as having uncontrolled asthma and about one-quarter (24.6%) were partially controlled asthma. Dust (91.6%), exposure to incense, detergent and essence (86.8%), common cold (82%) and cold weather (79.6%) were the factors that trigger or exacerbate asthma attacks. Physical activity/exercise and cold weather were the commonest factors that significantly exacerbate asthma attacks particularly among children and were mostly uncontrolled. More than one-third (36.5%) of the asthmatic patients in the PHCCs were cared for by general practitioners.

Conclusion

Poor asthma control was observed in a high proportion of asthmatic children, adolescents and adults in the Makkah region and they were mostly from non-specialized PHCCs. The poor asthma control among the respondents affects their quality of sleep ( i.e., frequent awakening at night), recurrent absences from work and school, increased hospitalizations, emergency and unscheduled visits to the hospital.

## Introduction

Asthma is the most common respiratory disease and accounts for approximately 300 million cases worldwide and 180,000 deaths annually [1]. In Saudi Arabia, asthma is a major public health concern affecting more than two million people and ranks 19th in terms of disability-adjusted life years (DALYs) and 26th in deaths. The prevalence of asthma among Saudi adults was relatively low (4.05%) as compared to children (8%-25%) [2].

Asthma has a negative impact on the life of patients, their families, and the community, including lost days of work, absence from school, and poor quality of life, which can eventually lead to frequent emergency department visits, hospitalizations, and sometimes, to death [2,3].

Achieving asthma control is a result of an interaction between different variables related to the disease pattern, patient's and physicians’ knowledge and behavior [2]. However, difficult to control asthma can be considered as a result from a complex interaction of different variables, such as some disease-related factors (i.e., the presence of other comorbidities) or patient-related factors (i.e., adherence to treatment, wrong technique, and coping strategies) [2,4]. Thus, the assessment of the level of asthma control can serve as a basis for changes in the asthma treatment, whether in maintaining, stepping-up, or stepping it down according to current asthma guidelines.

Therefore, this study aimed to assess the control status of the asthmatic patients in selected Primary Health Care Centers (PHCCs) in the city of Makkah, Saudi Arabia and to identify factors associated with impaired asthma control in order to improve their quality of life.

## Materials and methods

This was a cross-sectional study conducted from March 1, 2016, to May 31, 2016, in selected PHCCs in the city of Makkah, Saudi Arabia. This study employed stratified random sampling technique in selecting five out of 47 PHCCs to get a representative sample of different geographic locations of the city. 

Patients aged four and above and diagnosed with asthma based on the Saudi Initiative for Asthma (SINA) were the sample population of this study. The sample size of 208 was determined by using the Raosoft software (Raosoft, Inc., Seattle, USA ), based on the 90% confidence level, a margin of error of ±5%, and an ability to detect the prevalence of uncontrolled asthma in Makkah (i.e., response distribution) of 0.66 [5]. 

This study utilized two questionnaires for data collection. The first questionnaire was designed by the researcher to determine the demographic characteristics of the respondents, factors that trigger asthma attacks, and the burden of asthma. The content and construct of the questionnaire were validated by two family medicine consultants and one epidemiology professor. The second questionnaire was the Arabic version of the Asthma Control Test (ACT) for adolescents and adult (i.e., ≥ 12 years old) which was validated by SINA [6]. This tool consists of five items which cover limitations of patient's activity, shortness of breath, the frequency of night symptoms, use of rescue medication, and a rating of overall control of the disease over the past four weeks. However, children at the age of four to 11 years, ACT-Arabic version from Ghafouri et al. was used [5]. This is a two-part questionnaire with a total of seven questions, which cover the same points in the adult tool. The first part was answered by the child and the second part was answered by for the caregiver.

In the adolescent and adult ACT, answer to every question has a corresponding score ranging from one to five. This gives sum scores ranging from five to 25 (higher scores indicate better asthma control). A score of < 16 was considered as uncontrolled asthma, 16 to 19 as partially controlled asthma, and ≥ 20 as controlled asthma. In the children ACT, however, the score was made up of the sum of scores of the two parts, ranging from zero to 27 (higher scores indicate better asthma control). A score of ≤ 19 was considered as uncontrolled asthma [7]. 

The researchers developed five teams of data collectors and assigned to each of the five PHCCs. Respondents were interviewed and explained the objectives of this study. Informed verbal and written consent was obtained from the respondents before the commencement of the interview and collection of data. All collected data were kept confidential and anonymous.

All statistical analyses employed IBM SPSS version 21 (IBM Corp, Armonk, USA). To compare the differences between groups’ qualitative variables, the Chi-square test was used. However, if the expected value of any cell was less than five, Fisher's exact test was used. A p-value ≤ 0.05 was set to indicate statistically significant differences.

## Results

This study was able to enroll and collect data from 167 respondents out of 208 calculated sample size (i.e., 80% response rate). Of the 167 respondents, 48 (28.7%) were children (aged <12 years) and 119 (71.3%) were adolescents and adults (aged 12 years or older). Table 1 shows that 30 (62.5%) of children respondents were males, while 68 (57.1%) of adolescents and adults respondents were females. Majority of the respondents were Saudi nationals, 42 (87.5%) children and 102 (85.7%) adolescents and adults. Approximately two-thirds of the respondents, 42 (35.3%), had a family monthly income below 8,000 Saudi Riyals (i.e., 2,100 US Dollars), and 41 (34.5%) with bachelor’s degree. Twenty-nine (60.4%) and 48 (40.3%) of the children and adolescent/adults guardians’ jobs were intellectual, respectively.

**Table 1:** Characteristics of respondents according to age group SR: Saudi Riyal

Characteristics	Age Group			
	Children (n =48)		Adolescents and adults (n =119)	
	No	%	No	%
Gender				
Male	30	62.5%	51	42.9%
Female	18	37.5%	68	57.1%
Nationality				
Saudi	42	87.5%	102	85.7%
Non-Saudi	6	12.5%	17	14.3%
Monthly income of the family				
< 3000 SR	14	29.2%	36	30.3%
3000 – 7999 SR	15	31.2%	42	35.3%
8000 – 14999 SR	11	22.9%	27	22.7%
15000 – 19999 SR	2	4.2%	9	7.6%
20000 or above SR	6	12.5%	5	4.2%
Education level				
Illiterate	0	0.0%	7	5.9%
under-school	22	45.9%	0	0.0%
Primary	26	54.1%	23	19.3%
Elementary	0	0.0%	17	14.3%
Secondary	0	0.0%	27	22.7%
Diploma	0	0.0%	4	3.4%
Bachelor	0	0.0%	41	34.5%
Job of the guardian				
Unemployed	6	12.5%	32	26.9%
Manual / Field	13	27.1%	39	32.8%
Intellectual	29	60.4%	48	40.3%
Residency				
Alzaher-North	11	22.9%	24	20.2%
Alzahraa-West	7	14.6%	26	21.8%
Alaziziah-East	9	18.8%	24	20.2%
Almansor-Central	10	20.8%	21	17.6%
Al-Hijrah-South	11	22.9%	24	20.2%

**Figure 1:**
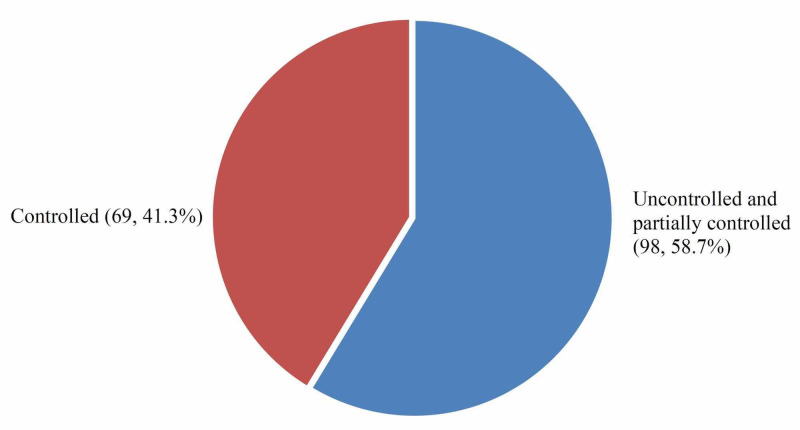
Respondents' level of asthma control

It is worth mentioning that 98 (58.6%) of the respondents had impaired asthma control and 69 (41.3%) had fully controlled asthma as shown in Figure 1. 

**Figure 2:**
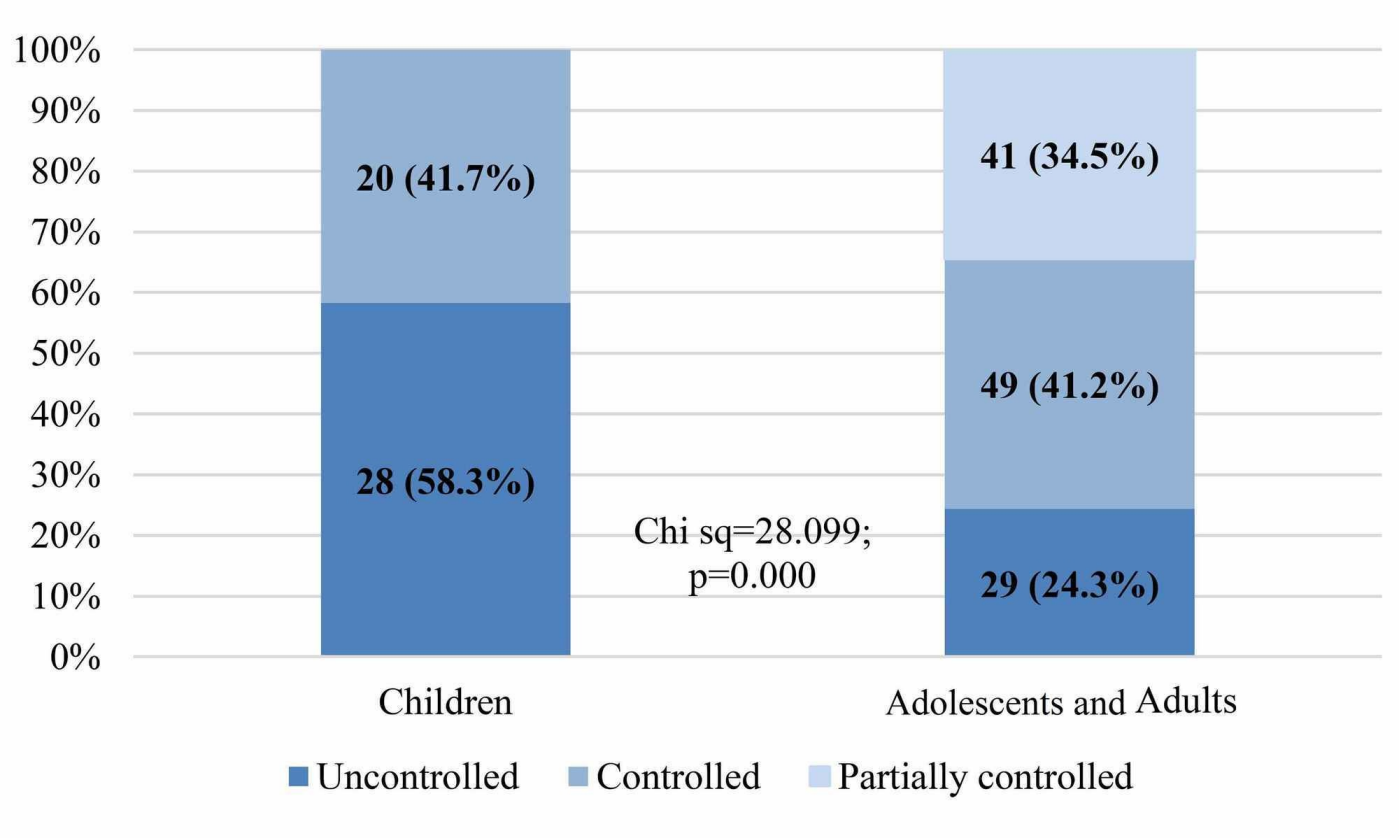
Level of asthma control by age category

When the data were analyzed according to age category, Figure 2 shows that respondents with uncontrolled asthma were significantly higher in children as compared to adolescents and adults (58.3% vs. 24.3%) p<0.05.

Table 2 shows that uncontrolled asthma among children was higher among males (66.7%), non-Saudis (66.7%), preschoolers (63.6%), with guardians who are employed in manual/field jobs (69.2%) and with a family income of 3,000 to 8,000 Saudi Riyals (73.3%). The highest frequency of uncontrolled asthma among children was observed in Almansor district (80%), whereas the lowest frequency was observed in Alzaher district (27.3%).

**Table 2:** Characteristics of asthmatic children according to the level of asthma control * Based on Chi-square test; SR: Saudi Riyal

Characteristics	Level of control					
	Uncontrolled		Controlled		X^2^	p*
	No	%	No	%		
Gender					2.286	0.131
Male	20	66.7%	10	33.3%		
Female	8	44.4%	10	55.6%		
Nationality					Fisher	0.658
Saudi	24	57.1%	18	42.9%		
Non-Saudi	4	66.7%	2	33.3%		
Monthly income					5.987	0.050
<3000 SR	10	71.4%	4	28.6%		
3000-<8000 SR	11	73.3%	4	26.7%		
8000+ SR	7	36.8%	12	63.2%		
Education level					0.470	0.493
Pre-school	14	63.6%	8	36.4%		
Primary	14	53.8%	12	46.2%		
Job of the guardian					1.328	0.515
Unemployed	4	66.7%	2	33.3%		
Manual/field	9	69.2%	4	30.8%		
Intellectual	15	51.7%	14	48.3%		
Residency					NA	NA
Alzaher-North	3	27.3%	8	72.7%		
Alzahraa-West	5	71.4%	2	28.6%		
Alaziziah Sh-East	5	55.6%	4	44.4%		
Almansor-Central	8	80.0%	2	20.0%		
Al-Hijrah-South	7	63.6%	4	36.4%		

As shown in Table 3, both uncontrolled and partially controlled asthma among adolescents and adult patients were higher in males than females. Uncontrolled asthma was mostly observed among Saudi nationals (27.5%), those with a family monthly income of <3,000 Saudi Riyals (30.6%), with an unemployed guardian (37.5%), and living in Alzahraa district (42.3%). 

**Table 3:** Characteristics of asthmatic adolescents and adults patients according to the level of asthma control * Based on Chi-square test; SR: Saudi Riyal

Characteristics	Level of Control							
	Uncontrolled		Controlled		Partially controlled		X^2^	p*
	No.	%	No.	%	No.	%		
Gender								
Male	13	25.5%	15	29.4%	23	45.1%	5.981	0.050
Female	16	23.5%	34	50.0%	18	26.5%		
Nationality								
Saudi	28	27.5%	38	37.3%	36	35.3%	5.595	0.061
Non-Saudi	1	5.9%	11	64.7%	5	29.4%		
Monthly income								
<3000 SR	11	30.6%	11	30.6%	14	38.9%	4.048	0.400
3000-<8000 SR	8	19.0%	22	52.4%	12	28.6%		
8000+ SR	10	24.4%	16	39.0%	15	36.6%		
Education level								
Illiterate	2	28.6%	2	28.6%	3	42.9%	NA	NA
Primary	7	30.4%	7	30.4%	9	39.1%		
Elementary	1	5.9%	10	58.8%	6	35.3%		
Secondary	5	18.5%	9	33.3%	13	48.1%		
Diploma/Bachelor	14	31.1%	21	46.7%	10	22.2%		
Job of the guardian								
Unemployed	12	37.5%	11	34.4%	9	28.1%	4.761	0.313
Manual/field	6	15.4%	18	46.2%	15	38.5%		
Intellectual	11	22.9%	20	41.7%	17	35.4%		
Residency								
Alzaher-North	5	20.8%	10	41.7%	9	37.5%	12.093	0.147
Alzahraa-West	11	42.3%	7	26.9%	8	30.8%		
Alaziziah Sh-East	3	12.5%	12	50.0%	9	37.5%		
Almansor-Central	2	9.5%	9	42.9%	10	47.6%		
Al-Hijrah-South	8	33.3%	11	45.8%	5	20.8%		

**Figure 3:**
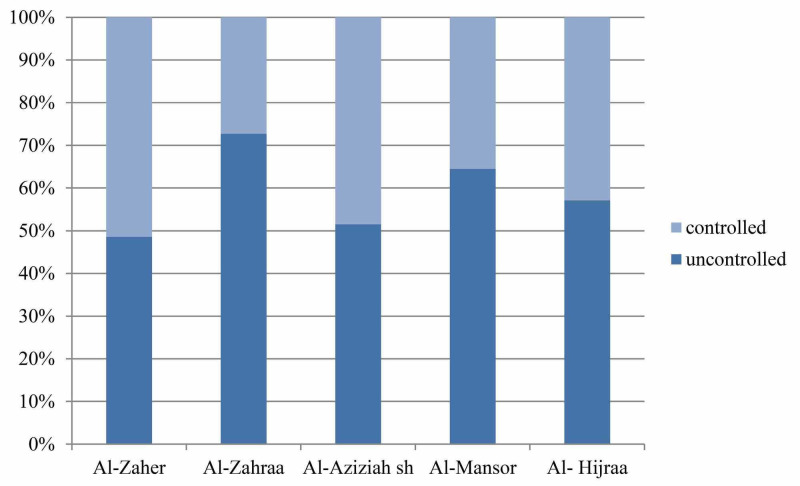
Comparison of respondents asthma control between PHCCs (Alzaher, Alzahraa, Alaziziah, Almansor and Alhijraa) PHCCs: primary health care centers

Figure 3 shows that non-specialized PHCCs (Alzahraa and Almansor) had higher percentages of respondents with impaired asthma control than in specialized PHCCs (Alaziziah, Alhijraa and Alzaher).

**Figure 4:**
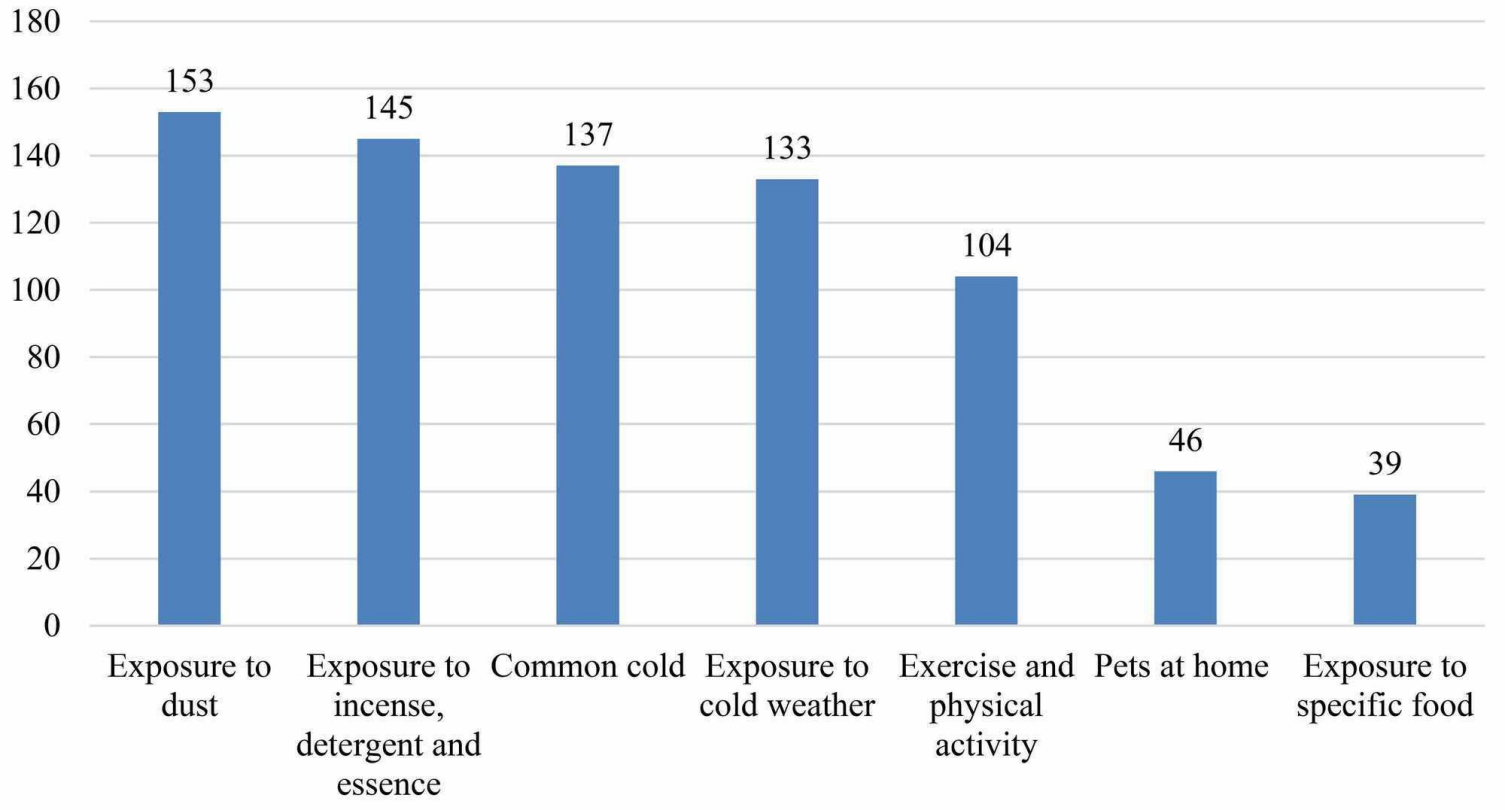
Factors that triggers or exacerbate asthmatic attacks

Figure 4 demonstrates that respondents exposure to dust was the commonest triggering factor of asthma attacks 153 (91.6%) followed by exposure to incense, detergent and essence 145 (86.8%). Common cold 137 (82%) and exposure to cold weather 133 (79.6%) were also reported by the considerable proportion of the respondents. Exposure to pets at home 49 (27.5%) and exposure to specific types of food 39 (23.4%) were the least triggering factors of asthma attacks.

Table 4 shows that among the children respondents, exposure to cold weather (p=0.003) and physical activity/exercise (p=0.041) were significantly associated with uncontrolled asthma. 

**Table 4:** Asthma control among children according to factors that trigger or exacerbate asthma attacks (n=48) * Based on Chi-square test ** statistically significant

Factors that exacerbate asthma attacks (number of patients answered yes )	Level of control					
	Uncontrolled		Controlled		X^2^	p*
	No	%	No	%		
Exposure to incense, detergent and essence (43)	26	92.9%	17	85.0%	0.772	0.380
Exercise and physical activity (34)	23	82.1%	11	55.0%	4.160	0.041**
Pets at home (13)	8	28.6%	5	25.0%	0.075	0.784
Exposure to dust (44)	27	96.4%	17	85.0%	1.995	0.158
Exposure to cold weather (42)	28	100%	14	70.0%	Fisher	0.003**
Common cold (44)	27	96.4%	17	85.0%	Fisher	0.294
Exposure to specific food (10)	5	17.9%	5	25.0%	Fisher	0.548
Smoker at home					1.626	0.202
Yes	12	42.9%	5	25.0%		
No	16	57.1%	15	75.0%		
Family history of allergy					Fisher	0.348
Yes	24	85.7%	15	75.0%		
No	4	14.3%	5	25.0%		
Training by expertise					1.829	0.176
Yes	23	82.1%	13	65.0%		
No	5	17.9%	7	35.0%		
Correct use of inhaler					NA	NA
Yes	23	82.1%	13	65.0%		
No	2	7.1%	4	20.0%		
Not sure	3	10.7%	3	15.0%		

Table 5 illustrates that a higher percentage of adolescents and adult patients with uncontrolled asthma was exacerbated by exposure to dust (96.6%), incense, detergent and essence (89.7%), and cold weather (86.2%). However, respondents with uncontrolled, controlled and partially controlled asthma were no significant difference in terms of factors exacerbating asthma attacks.

**Table 5:** Asthma control of adolescents and adult respondents according to factors that trigger or exacerbate asthma attacks * Based on Chi-square test

Factors that exacerbate asthma attacks (number of patients answered yes )	Level of Control							
	Uncontrolled		Controlled		Partially controlled			
	No.	%	No.	%	No.	%	X^2^	p*
Exposure to incense, detergent and essence (102)	26	89.7%	40	81.6%	36	87.8%	1.181	0.554
Exercise and physical activity (70)	20	69.0%	24	49.0%	26	63.4%	3.549	0.170
Pets at home (33)	5	17.2%	14	28.6%	14	34.1%	2.451	0.294
Exposure to dust (109)	28	96.6%	45	91.8%	36	87.8%	1.695	0.429
Exposure to cold weather (91)	25	86.2%	36	73.5%	30	73.2%	2.021	0.364
Common cold (93)	24	82.8%	38	77.6%	31	75.6%	0.526	0.769
Exposure to specific food (29)	10	34.5%	9	18.4%	10	24.4%	2.567	0.277
Smoking status								
Yes	9	31.0%	6	12.2%	11	26.8%	4.675	0.097
No	20	69.0%	43	87.8%	30	73.2%		
Smoker at home								
Yes	13	44.8%	25	51.0%	17	41.5%	0.850	0.654
No	16	55.2%	24	49.0%	24	58.5%		
Family history of allergy								
Yes	23	79.3%	40	81.6%	30	73.2%	0.966	0.617
No	6	20.7%	9	18.4%	11	26.8%		
Training by expertise								
Yes	23	79.3%	41	83.7%	32	78.0%	0.499	0.779
No	6	20.7%	8	16.3%	9	22.0%		
Correct use of inhaler								
Yes	23	79.3%	41	83.7%	32	78.0%	NA	NA
No	5	17.2%	5	10.2%	7	17.1%		
Not sure	1	3.4%	3	6.1%	2	4.9%		

**Figure 5:**
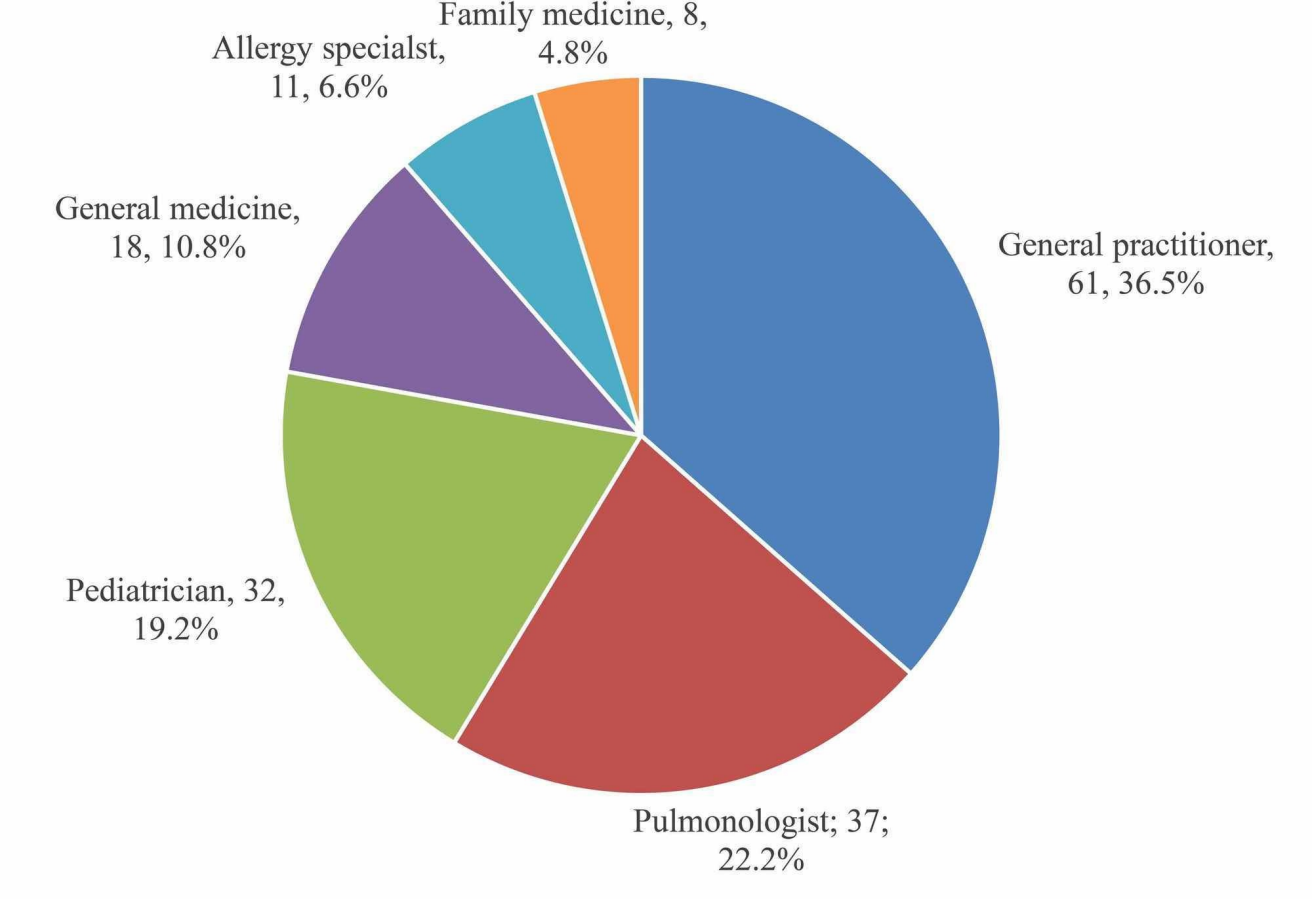
Treating physicians for asthma

In Figure 5 shows that 36.5% of asthmatic patients in the PHCCs were cared for by general practitioners, 22.2% by pulmonologist, and 19.2% by pediatricians. Only 4.8% of the asthmatic patients were cared for by family medicine specialists.

In Table 6, most of the controlled asthmatic patients (70%) and more than one-half of the uncontrolled asthmatic children (53.6%) were cared by a pediatrician, and only one child was uncontrolled (3.6%) and treated by family medicine practitioner.

**Table 6:** Level of asthma control of children according to treating physician * Based on Chi-square test

Treating physician	Level of control					
	Uncontrolled		Controlled		X^2^	p*
	No.	%	No.	%		
Family medicine	1	3.6%	0	0.0%	NA	NA
General practitioner	4	14.3%	4	20.0%		
Pediatrician	15	53.6%	14	70.0%		
General medicine	0	0.0%	1	5.0%		
Allergy specialist	2	7.1%	0	0.0%		
Pulmonologist	6	21.4%	1	5.0%		

In Table 7, more than one-half of the partially controlled adolescents and adult asthmatic patients (56.1%) and 38.8% of the controlled and 37.9% of the uncontrolled patients were treated by general practitioners.

**Table 7:** Level of asthma control of adolescents and adults patients according to treating physician * Based on Chi-square test

Treating physician	Level of control							
	Uncontrolled		Controlled		Partially controlled		X^2^	p*
	No.	%	No.	%	No.	%		
Family medicine	1	3.4%	3	6.1%	3	7.3%		
General practitioner	11	37.9%	19	38.8%	23	56.1%		
Pediatrician	0	0.0%	1	2.0%	2	4.9%	NA	NA
General medicine	6	20.7%	8	16.3%	3	7.3%		
Allergy specialist	3	10.3%	3	6.1%	3	7.3%		
Respirologist	8	27.6%	15	30.6%	7	17.1%		

After excluding the preschool-age children, Table 8 shows that the absenteeism among school children was higher among uncontrolled asthmatic patients (81%) than the controlled (58.8%). Uncontrolled asthmatic children were more likely to be admitted due to asthma (17.9%) than the controlled (10%), however, these differences were not statistically significant (p>0.05). Three-quarters of the controlled asthmatic children (75%) never wake up during the night due to asthma attack compared to none of the uncontrolled as all of them experienced waking up at night due to asthma either one to three nights weekly (42.9%) or four or more nights weekly (57.1%) which were statistically significant.

**Table 8:** Burden of asthma attacks on asthmatic children according to the level of asthma control * Based on Chi-square test; ** Statistically significant ER: emergency room; PHC: primary health center

Characteristics	Level of control					
	Uncontrolled		Controlled		X^2^	p*
	No	%	No	%		
Absenteeism (n=38):					Fisher	0.167
Yes	17	81.0%	10	58.8%		
No	4	19.0%	7	41.2%		
Admission due to asthma during the past year:					Fisher	0.683
Yes	5	17.9%	2	10.0%		
No	23	82.1%	18	90.0%		
Frequent visits to ER or PHC during last year:					0.075	0.784
Yes	20	71.4%	15	75.0%		
No	8	28.6%	5	25.0%		
Wake up during night due to asthma attack:					31.785	<0.001**
Never	0	0.0%	15	75.0%		
1-3 nights weekly	12	42.9%	4	20.%		
4+ nights weekly	16	57.1%	1	5.5%		

The findings in Table 9 show that absenteeism was mostly reported among adolescents and adult patients with partially controlled (68.6%) and uncontrolled asthma (45.8%) than to those with controlled asthma (34.1%), which was statistically significant (p<0.05). In addition, these respondents with uncontrolled (72.4%) and partially controlled asthma (73.2%) were had frequent visits in the ER or PHCCs than respondents with controlled asthma (36.7%), which was also statistically significant (p<0.05). Respondents with controlled asthma (72%) were significantly never experienced waking up at night due to the asthma attack as compared to respondents with uncontrolled (6.9%) and partially controlled asthma (10%), p<0.05.

**Table 9:** Burden of asthma attacks on asthmatic adolescents and adults respondents according to the level of asthma control *Based on Chi-square test **Statistically significant

Characteristics	Level of control							
	Uncontrolled		Controlled		Partially controlled		X^2^	p*
	No.	%	No.	%	No.	%		
Absenteeism (n=100)								
Yes	11	45.8%	14	34.1%	24	68.6%	9.081	0.011**
No	13	54.2%	27	65.9%	11	31.4%		
Admission due to asthma during the past years								
Yes	4	13.8%	4	8.2%	8	19.5%	2.474	0.290
No	25	86.2%	45	91.8%	33	80.5%		
Frequent visits to ER or PHC during last year								
Yes	21	72.4%	18	36.7%	30	73.2%	15.441	<0.001**
No	8	27.6%	31	63.3%	11	26.8%		
Wake up during night due to asthma attack:								
Never	2	6.9%	36	72%	4	10.0%	73.912	<0.001**
1-2 nights monthly	4	13.8%	13	26%	20	50.0%		
1-3 nights weekly	18	62.1%	1	2.0%	14	35.0%		
4+ nights weekly	5	17.2%	0	0.0%	2	5.0%		

## Discussion

Asthma is a public health problem representing the commonest respiratory disease. This disease places significant health and economic burden to patients, families, and health systems. In Saudi Arabia, asthma is one of the most common chronic disease affecting about two million individuals and responsible for frequent emergency visits and mortalities [2,3]. The negative impact of asthma is exaggerated when uncontrolled. Due to these reasons, the current study comes in the path of exploring the level of control of asthma in patients attending the primary health care centers and to find out the possible factors associated with the impaired control.

Impaired asthma control among asthmatic patients was high in Makkah when compared to the controlled asthma patients and that what we observed in researches done in the different regions in the Kingdom of Saudi Arabia. The 58.7% of the asthmatic patients were had impaired asthma control whereas 41.3% had asthma control. The prevalence of uncontrolled asthmatic patients varies between different countries worldwide; our findings are close to most of the reported prevalence. For example, in Denmark, the prevalence of uncontrolled asthmatic adult patients was 30.8%, in Portugal the overall uncontrolled asthma among all age groups was 43.1%, in France, the control among asthmatic children was unacceptable in 41.2% [8,9,10]. On the other hand, the prevalence of impaired asthma control among asthmatic patients in Makkah (adolescent and adult 58.8% and children 58.3%) was the lowest when to compared to researches done in Riyadh (adult 95% and children 59.3% and Taif (88% impaired asthma in children and adolescent) [11,12,13].

An alarming finding was observed in the United States of America, as it was found that the prevalence of uncontrolled asthma had increased from 41% in 2007 to 47% in 2009 [14,15]. Also, we observed this increase in the prevalence of uncontrolled asthma among adult in Riyadh, KSA, from 64% 2006 to 68.1 in 2014 [11,16].

Apart from the differences in the prevalence of uncontrolled asthmatic patients observed in different researches that could be attributed to many factors such as differences in study settings, all are sharing the fact that the prevalence is remarkably high which necessitate vigorous investigation to find out its reasons. In this respect, Braido (2013) attributed the reasons for the failure of asthma control to factors grouped into three categories, disease-related, patient-related and doctor related [4]. In Saudi Arabia, Al-Moamary and his colleagues stated that the problem of uncontrolled asthma is multifactorial and related partly to poor knowledge of the primary health care physicians and their fear about using new drugs for control of asthma patients [2]. In this respect, Ghafouri addressed in 2011 that the percentage of uncontrolled asthmatic patients was highest in non-specialized PHCCs by 66% in Al-Kakiah than specialized PHCCs by 46% in Iskan [5]. In our study, non-specialized PHCCs showed the highest percentage of uncontrolled asthma by 72.7% in Al-Zahraa and 64.52% in Almansor. On the other hand, in specialized PHCCs showed different percentages of uncontrolled asthma 48.57% in Alzaher, 51.51% in Alaziziah and 57.14 % in Al-Hijraa.

According to the disease-related factors, Braido stated that the triggers of asthma involve house dust mite, pets, occupational exposure, exercise, drug, passive smoking, new allergens, aspirin, and beta-blockers [4]. In our study, the commonest factors triggers to exacerbate asthma attack among our patients were exposure to dust, incense, detergent, and essence. Although exposure to pets at home was found as the least common factor that triggers asthma attack, however, raising pets such as dogs or cats were being discourage.

Many other factors influencing the level of control of asthma had been reported in researches; among these factors, the socioeconomic status which plays an important role. Low economic level is usually associated with poor environmental conditions which worsen asthma and increase the liability for severe asthma attacks [17]. Wright and his colleague (2003) mentioned that patients with lower economic status have higher exposure to indoor allergens such as dust, cockroaches, and tobacco smokes as well as outdoor allergens such as pollutants [18]. These allergens are responsible for provoking and exaggerating asthma particularly among children [19]. This might explain our findings where asthmatic children living in families with lower monthly income had the highest likelihood of being uncontrolled. Also, the similar result of low economic level and uncontrolled asthma observed in multiple researches done in Riyadh [11,12,13].

Sex hormones are believed to play a role in the development and outcome of asthma, while asthma is more prevalent among boys than girls, the reverse occurs after puberty; this gender susceptibility is attributed to hormonal differences and sex-specific variance in environmental exposure [20]. A USA national asthma survey reports that women had poor asthma control [21]. But in our study, there was no statistically significant gender difference in the level of control among children, but there was a borderline difference in adolescents and adults, where the uncontrolled and partially controlled asthmatic were more frequent among males than females. We observed that our result differed from Ghafouri article which published in 2011 which showed that the uncontrolled asthmatics were more frequent among female than male [5]. This difference could be due to the relatively small study sample size as compared to our study. Hawthorne and his colleagues published a study in 2008 reported that females tend to follow their prescriptions better than males [22]. Although, previous study has suggested that female was considered as a risk factor for worsening asthma, however, in the recent study reported that male have worse asthma control than female based on the level of b2-agonist use [21,23].

In our study, the exercise-induced asthma was significantly higher in uncontrolled than controlled asthmatic children. In this respect, Carlsen, addressed in 2002 that physical exercise is associated with increased ventilation with subsequent heat and water loss, which initiate release of mediators that stimulate airways receptors with subsequent bronchospasm in asthmatic children [24]. In the same context, the Asthma and Allergy Foundation of America cited that cold air cause bronchoconstriction, which is when the airways narrow, causing breathing to become different and stressful [25]. Accordingly, it was noticed in our study that exposure to cold weather was a significant triggering factor for asthmatic attack among all asthmatic children. The similar result was observed in Taif research among asthmatic children [13].

Asthmatic patients are bothered by symptoms which impair their lives. Symptoms include shortness of breath, tightness of chest and cough which usually exacerbated at night, these symptoms sometimes deprive patients from having good sleep or even awakening them frequently at night [4]. In a multinational survey in seven European countries covering 2800 asthmatic patients, asthma-related sleep disturbances at least once a week was detected in 30% of the patients [26]. In our study, there was a significantly higher proportion of the uncontrolled than controlled asthmatic patients frequently waking up at night due to asthmatic attack.

The same multinational survey showed that, among the investigated 2800 asthmatic patients, one quarter had unscheduled urgent care visits and 10% had one or more emergency room visits while 7% had overnight hospitalization due to asthma, that was asserted by Chapman and his colleagues in 2008 who said that poor daily control is associated with more hospitalizations, emergency and unscheduled visits, and other healthcare contacts [26,27]. In our study, if compared to controlled patients, a significantly higher proportion of the uncontrolled (72.4%) and partially controlled patients (73.2%) had frequent ER or PHCCs visits in the past year.

As consequences of frequent poor sleep and need for health care, absenteeism is common in asthmatic patients in general and among uncontrolled ones in particular [28]. In our patients, significant high percentage of the uncontrolled (45.8%) and partially controlled adolescent and adult patients (68.6%) reported that they had frequent absenteeism due to asthma. Our results were in accordance with what was found in the two-year study in USA evaluating approximately 4,000 patients and showed that uncontrolled asthma had higher annual mean number of work days lost compared with patients who had controlled asthma (7.1 vs 0.4) [29].

Finally, the current study showed that more than one third of the asthmatic patients in the primary health care centers are cared by general practitioners (36.5%) who represent the majority of the physicians working in PHCCs, which come in congruence with what was found in a study carried out in USA where 29% of the asthmatic patients prefer to be treated by general practitioners, as they observed that the patients who preferred to visit allergist or pulmonologist had poorly controlled asthma than well controlled or partially controlled [15]. That could be justified by the severity of cases who are usually referred to the pulmonologist or allergist rather than being an outcome for the patients treated by these specialists.

## Conclusions

Poor asthma control was observed in a high proportion of asthmatic children, adolescents, and adults in the Makkah region and they were mostly from non-specialized PHCCs. Physical activity/exercise and cold weather were the commonest factors that trigger or exacerbate asthma attacks particularly among children and these were mostly uncontrolled. The poor asthma control affects respondents quality of sleep (i.e., frequent awakening at night due to asthmatic attack), recurrent absences from work and school, increased hospitalizations, emergency and unscheduled visits to the hospital. More than one-third of the asthmatic patients in PHCCs were cared for by general practitioners. Further research should focus on determining the prevalence of increasing uncontrolled asthma in urban communities, and also assess their quality of life. Lastly, this study is further suggested to assess the knowledge of general practitioners in PHCCs about asthma management.
